# Whole-Body Human Inverse Dynamics with Distributed Micro-Accelerometers, Gyros and Force Sensing †

**DOI:** 10.3390/s16050727

**Published:** 2016-05-20

**Authors:** Claudia Latella, Naveen Kuppuswamy, Francesco Romano, Silvio Traversaro, Francesco Nori

**Affiliations:** iCub Facility Department at Istituto Italiano di Tecnologia, Via Morego 30, Genova, Italy; claudia.latella@iit.it (C.L.); naveen.kuppuswamy@iit.it (N.K.); francesco.romano@iit.it (F.R.); silvio.traversaro@iit.it (S.T.)

**Keywords:** whole-body force tracking, human wearable dynamics, human inverse dynamics

## Abstract

Human motion tracking is a powerful tool used in a large range of applications that require human movement analysis. Although it is a well-established technique, its main limitation is the lack of estimation of real-time kinetics information such as forces and torques during the motion capture. In this paper, we present a novel approach for a human soft wearable force tracking for the simultaneous estimation of whole-body forces along with the motion. The early stage of our framework encompasses traditional passive marker based methods, inertial and contact force sensor modalities and harnesses a probabilistic computational technique for estimating dynamic quantities, originally proposed in the domain of humanoid robot control. We present experimental analysis on subjects performing a two degrees-of-freedom bowing task, and we estimate the motion and kinetics quantities. The results demonstrate the validity of the proposed method. We discuss the possible use of this technique in the design of a novel soft wearable force tracking device and its potential applications.

## 1. Introduction

Human whole-body motion tracking is nowadays a well-established tool in the analysis of human movements. This tool has found a wide variety of real-world applications ranging from entertainment (movies and games) to sport and rehabilitation frameworks. Several commercial solutions for whole-body motion tracking are available. Well known examples include: a wearable marker-less technology suitable for outdoor motion capturing produced by Xsens [1] (Xsens Technologies B.V., Enschede, Netherlands), a state-of-the-art marker-based technology for in-lab applications produced by Vicon (Vicon Motion Systems Ltd, Oxford, UK) and Microsoft Kinect depth camera system (Microsoft Corporation, Redmond, Washington), which allows marker-less low-cost whole-body motion tracking for indoor applications [2]. In biomechanics, soft wearable and stretchable systems have been proposed for measuring human body motion [3,4], as well as in health activity monitoring [5]. Combinations of different technologies have also been used for detecting human motion: in [6], a video-based motion technique was adopted for capturing realistic human motion from video sequences. Although existing technologies provide a high level of accuracy in computing motion quantities, they have several limitations in their ability to measure kinetic quantities in real-time (kinetics considers forces that cause movements). A key problem lies in the fact that motion capture methods typically employ only kinematic measurement modalities (position, velocities and accelerations) [7] and do not include information on the kinetics of human movements.

The importance of including and exploiting all dynamic information is a crucial point in several research areas such as ergonomics for industrial scenarios, developing prosthetic devices and exoskeleton systems in rehabilitation fields, or in human-robot interaction. For these reasons, whole-body force tracking is not a new challenge for the scientific community, but the topic has been seldom explored *in situ* due to the computational difficulties of the analysis and even more rarely analyzed in real-time modality. Although several recent studies are going in this direction, it is limited to prototypical and non-wearable technologies. Typical non-wearable technologies involve the combination of a motion capture system (e.g., a Vicon camera system ), with commercial force plates and Newtonian physics to perform inverse dynamics computations . Some recent prototypes [8] have been proposed for kinematics and dynamics motion capture. The suggested approach involves a Kinect-like sensing and pressure-sensing shoes to reconstruct the whole-body dynamics.

To obtain forces and torques acting on a body, accurate data on the mass, center of mass and inertias of each body segment are needed. Different methods for the determination of body segment parameters can be described in [9], but the real difficulty is that a universal acknowledged procedure for estimating inertial parameters with accuracy does not exist yet. In this framework, it is fundamental to specify exactly what the kinematics and external boundaries are.

Towards compensating for aforementioned drawbacks, one approach is to supplement or even replace standard marker-based technologies in several applications with a system design embedding a combination of Inertial Measurement Unit (IMU) sensors and force sensing for evaluating contact forces. However, in order to properly model and understand the role of dynamic quantities, an appropriate understanding of the dynamic interaction between the elements of the models (such as reaction and contact forces, accelerations of links and forces exchanged between them) is needed.

Inspired by a recent research study on sensor fusion for whole-body estimation on the humanoid iCub robot [10] (Istituto Italiano di Tecnologia, Genova, Italy), in this paper, we propose a novel framework for wearable dynamics (*WearDY*) aiming to bridge the gap in dynamics analysis by fusing motion and force capture. The novelty of the approach consists of framing computations in a probabilistic Gaussian framework in the presence of redundant (and noisy) measurements. In this way, sensors play an *active* role in the computation since the classical boundary condition in recursive Newton–Euler algorithms (*i.e.*, linear-angular velocities and acceleration at the base link and forces–torques at the end-effector) are replaced with measurements coming from sensors. *WearDY* is an attempt at combining dynamic computations with stochastic estimation of dynamics variables. The early stage of *WearDY* tool encompass different modules: (1) a motion capture system computing human joint model configuration and kinematics; (2) an additional inertial sensor; (3) force platform sensing; and (4) a probabilistic algorithm framework. The final prototype will replace the motion capture system with a soft wearable suit embedding sensing structure.

In this paper, we focus mainly on describing the fourth module and its integration into the physical tool. We test the *WearDY* prototype on human subjects performing a two Degrees-of-Freedom (DOF) bowing task. The subjects are equipped with the basic elements of the proposed suit in the form of a chest-mounted IMU, a conventional motion capture system along with a force plate. The Gaussian algorithm is then applied to compute joint torques as well as other dynamic quantities such as link accelerations and transmission forces throughout the motion. The results demonstrate the applicability of the proposed method for the simultaneous force and motion tracking in dynamic motion.

The paper is structured as follows. Section 2 presents an overview of some important definitions of the algebraic notation used for computations along with the assumptions on the dynamic model. Section 3 provides an analytic statement of dynamic analysis wherein the probabilistic computation is framed. In Section 4, the probabilistic estimation theory is shown. The experimental analysis on human subjects is presented in Section 4 followed by concluding remarks in Section 4.

## 2. Notation

### 2.1. Spatial Algebra Description

**Figure 1:**
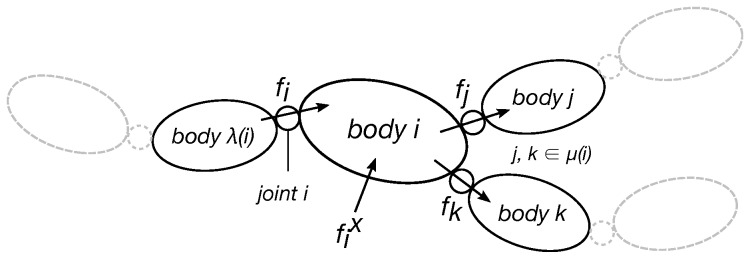
Graphical representation of an articulated rigid body as an oriented kinematic tree.

The notation adopted in *WearDY* architecture mirrors that in [11] (readers already familiar with the notation can jump to Section III): all variables are spatial vectors (six dimensional vectors including angular quantities in the first three components and the rest as linear quantities). Within this notation, an articulated rigid body is a system modeled as an oriented kinematic tree (Figure 1) with $N_{B}$ moving links numbering from 1 to $N_{B}$ (0 is the fixed base). Each link in the model is associated with a unique node in the tree. Node numbers can be always selected in a topological order so that each node *i* has a higher number than its unique parent λ(i) and a smaller number than all the nodes in the set of its children μ(i). Links *i* and its parent λ(i) are coupled with joint *i* according to Denavit–Hartenberg convention for joint numbering [12]. Joint *i* motion freedom subspace is modeled with $S_{i}\in R^{6\times n_{i}}$, being $n_{i}$ the number of DOF of the joint *i* and $n=n_{1}+\ldots +n_{N_{B}}$ the total number of DOFs of the system excluding the fixed base. For each link *i* and joint *i*, the following spatial quantities are defined:

$v_{i}$ velocity of link *i*;
$a_{i}$ acceleration of link *i*;
$f_{i}$ force transmitted from λ(i) to body *i*;
$f_{i}^{B}$ net force on link *i*;
$f_{i}^{x}$ external force acting on link *i*;
$q_{i}$ joint *i* position;
$\dot{q_{i}}$ joint *i* velocity;
$\ddot{q_{i}}$ joint *i* acceleration;
$\tau_{i}$ joint *i* torque;
$v_{Ji}$ velocity across joint *i*.

All variables are expressed in body *i* coordinates, except for $f_{i}^{x}$ which is convenient to express in absolute (*i.e.*, body 0) coordinates. To each link *i*, a spatial inertia tensor is also associated:

$$I_{i}=\left[\begin{array}{cc} I_{C,i}+m_{i}c_{i}\times c_{i}^{⊤} & m_{i}c_{i}\times \\ m_{i}c_{i}\times^{⊤} & m_{i}I_{3\times 3} \end{array}\right],$$

where $I_{C,i}$ is the spatial inertia tensor with respect to (w.r.t.) the link center of mass, $m_{i}$ is the total mass, $c_{i}$ is the relative displacement between the center of mass and the origin of the reference frame associated to the link. Within this spatial framework, in the paper, the following operations are adopted: × is the motion cross product operator such as, if

$$r=\left[\begin{array}{c} r_{x}\\ r_{y}\\ r_{z} \end{array}\right]\;\Rightarrow \;r\times =\left[\begin{array}{c} r_{x}\\ r_{y}\\ r_{z} \end{array}\right]\times =\left[\begin{array}{ccc} 0 & -r_{z} & r_{y}\\ r_{z} & 0 & -r_{x}\\ -r_{y} & r_{x} & 0 \end{array}\right].$$

Its dual operator $\times^{*}$ is the force cross product operator. The motion vector transformation ${}^{B}X_{A}$ from *A* to *B* coordinates and its analogous transformation for a force vector ${}^{B}X_{A}^{*}$ are, respectively:

$${}^{B}X_{A}=\left[\begin{array}{cc} {}^{B}R_{A} & 0_{3\times 3}\\ {-}^{B}R_{A}{(}^{A}r\times ) & {}^{B}R_{A} \end{array}\right],{\;}^{B}X_{A}^{*}=\left[\begin{array}{cc} {}^{B}R_{A} & {-}^{B}R_{A}{(}^{A}r\times )\\ 0_{3\times 3} & {}^{B}R_{A} \end{array}\right].$$

A homogeneous transformation matrix from *A* to *B* is also described as follows:

$${}^{B}T_{A}=\left[\begin{array}{cc} {}^{B}R_{A} & {-}^{B}R_{A}{(}^{A}r)\\ 0 & 1 \end{array}\right].$$

Throughout the paper, we denoted with ${}^{A}\dot{x}$ the temporal first order derivative and with ${}^{A}\ddot{x}$ the temporal second order derivative of a generic vector ***x*** in *A* coordinates.

### 2.2. Stochastic Notation

Given a stochastic variable ***x***, we denote with p(x) its probability density and with p(x|y) the conditional probability density of ***x*** given the assumption that another stochastic variable *****y***** has occurred. Since *****y***** is associated to a deterministic function f(x), with $E_{x}\left[f\left(x\right)\right]$ we denote the expected value of f(x) w.r.t. the probability distribution p(x). With $\mu_{x}$, $\Sigma_{x}$, we denote the mean and covariance of ***x***, *i.e.*, $\mu_{x}=E\left[x\right]$ and $\Sigma_{x}=E\left[xx^{⊤}\right]$. The probability density function of a multivariate Gaussian distribution $x\in R^{n}$ is $p\left(x\right)∼N\left(\mu_{x},\Sigma_{x}\right)$:

$$p\left(x\right)={\left(2\pi \right)}^{-\frac{n}{2}}{\left|\Sigma_{x}\right|}^{-\frac{1}{2}}\exp\left\{-\frac{1}{2}{\left(x-\mu_{x}\right)}^{⊤}\Sigma_{x}^{-1}\left(x-\mu_{x}\right)\right\},$$

where $\left|\Sigma_{x}\right|$ denotes the determinant of the matrix $\Sigma_{x}\in R^{n\times n}$. It is worth to notice that when in a multivariate normal distribution the covariance Σ is not a full-rank matrix, then the distribution is degenerate and does not have a density. In order to avoid the problem, it can be useful restrict the problem on a subset of Σ such that the covariance matrix for this subset is positive definite.

## 3. Problem Statement and Formulation

The dynamic estimation algorithm has been originally developed in [10] as a framework for the probabilistic estimation of whole-body robot dynamics with redundant measurements. The methodology was here adapted to fit the needs of the human motion. The present section discusses the estimation problem in details. After discussing the recursive Newton–Euler algorithm for inverse dynamics computation in Section 3.1, we arrange the resulting equations in a matrix formulation (Section 3.2). Section 3.3 introduces the estimation problem by discussing the case in which the boundary conditions of the Newton–Euler algorithm are replaced with a set of redundant measurements expressed in a new equation form.

### 3.1. Recursive Newton–Euler Algorithm

In [11], the inverse dynamics problem is formulated as the problem of finding the forces required to produce a given acceleration. It can be summarized by the following function:

$$\begin{array}{c} \tau =\mathrm{InvD}(model,q,\dot{q},\ddot{q},f^{x}) \end{array}$$

In biomechanics literature, different inverse dynamics approach are used [13]. Let us assume use of a "top-down" approach. We will assume that all quantities depending on ***q*** and $\dot{q}$ have been precomputed, including the transformation matrices ${}^{j}X_{i}$, ${}^{j}X_{i}^{*}$ and the velocities $v_{Ji}$, $v_{i}$ which can be efficiently computed with the following recursive equation:

(1a) $$\begin{array}{ccc} v_{Ji} & = & S_{i}\dot{q_{i}} \end{array}$$

(1b) $$\begin{array}{ccc} v_{i} & = & {}^{i}X_{\lambda (i)}v_{\lambda (i)}+v_{Ji.} \end{array}$$

A classical efficient numerical solution of inverse dynamics problem is given by the recursive Newton–Euler algorithm (RNEA) consisting of the following steps, expressed in body *i* coordinates:

(2a) $$\begin{array}{ccc} a_{i} & = & {}^{i}X_{\lambda (i)}a_{\lambda (i)}+S_{i}{\ddot{q}}_{i}+v_{i}\times v_{Ji} \end{array}$$

(2b) $$\begin{array}{ccc} f_{i}^{B} & = & I_{i}a_{i}+v_{i}\times^{*}I_{i}v_{i} \end{array}$$

(2c) $$\begin{array}{ccc} f_{i} & = & f_{i}^{B}-{}^{i}X_{0}^{*}f_{i}^{x}+\sum_{j\in \mu (i)}{}^{i}X_{j}^{*}f_{j} \end{array}$$

(2d) $$\begin{array}{ccc} \tau_{i} & = & S_{i}^{⊤}f_{i} \end{array}$$

Equations (1a), (1b) and (2a) are propagated from i=1 to $N_{B}$ with initial conditions $v_{0}=0$ and $a_{0}=-a_{g}$, which corresponds to the gravitational spatial acceleration vector expressed in the body frame 0 (null in its first three components and equal to the gravitational acceleration in the last three). Equations (2b)–(2d) are propagated from $i=N_{B}$ to 1.

### 3.2. RNEA Matrix Formulation and the Measurements Equation

In this section, a matrix arrangement of the RNEA is presented. Equation (2) can be seen as a set of equations which the below listed *dynamic variables* have to satisfy. Let us first define a spatial vector *****d***** of dynamic variables as follows:

(3a) $$\begin{array}{c} d={\left[\begin{array}{cccc} d_{1}^{⊤} & d_{2}^{⊤} & \cdots & d_{N_{B}}^{⊤} \end{array}\right]}^{⊤}\in R^{24N_{B}+2n} \end{array}$$

(3b) $$\begin{array}{c} d_{i}={\left[\begin{array}{cccccc} a_{i}^{⊤} & {f_{i}^{B}}^{⊤} & f_{i}^{⊤} & \tau_{i} & {f_{i}^{x}}^{⊤} & {\ddot{q}}_{i} \end{array}\right]}^{⊤}\in R^{24+2n_{i}} \end{array}$$

Given Equation (3), Equation (2) can be compactly written in the following matrix equation:

(4) $$D\left(q,\dot{q}\right)d+b_{D}\left(q,\dot{q}\right)=0$$

where ***D*** is a block matrix $\in R^{(18N_{B}+n)\times d}$ and $b_{D}$ is a vector $\in R^{18N_{B}+n}$. Let us define how to build ***D*** matrix and $b_{D}$ vector:

$$\begin{array}{c} D=\left[\begin{array}{ccc} D_{1,1} & \cdots & D_{1,N_{B}}\\ \vdots & \ddots & \vdots \\ D_{N_{B},1} & \cdots & D_{N_{B},N_{B}} \end{array}\right],\;b_{D}=\left[\begin{array}{c} b_{1}\\ \vdots \\ b_{N_{B}} \end{array}\right]. \end{array}$$

In particular:

$$\begin{array}{c} D_{i,i}=\left[\begin{array}{cccccc} -1 & 0 & 0 & 0 & 0 & S_{i}\\ I_{i} & -1 & 0 & 0 & 0 & 0\\ 0 & 1 & -1 & 0 & -{}^{i}X_{0}^{*} & 0\\ 0 & 0 & S_{i}^{⊤} & -1 & 0 & 0 \end{array}\right],\\ \forall j\in \mu \left(i\right)\;D_{i,j}=\left[\begin{array}{cccccc} 0 & 0 & 0 & 0 & 0 & 0\\ 0 & 0 & 0 & 0 & 0 & 0\\ 0 & 0 & {}^{i}X_{j}^{*} & 0 & 0 & 0\\ 0 & 0 & 0 & 0 & 0 & 0 \end{array}\right],\;j=\lambda \left(i\right)\;D_{i,j}=\left[\begin{array}{cccccc} {}^{i}X_{\lambda (i)} & 0 & 0 & 0 & 0 & 0\\ 0 & 0 & 0 & 0 & 0 & 0\\ 0 & 0 & 0 & 0 & 0 & 0\\ 0 & 0 & 0 & 0 & 0 & 0 \end{array}\right],\\ \mathrm{if}\;\lambda \left(i\right)=0\;b_{i}=\left[\begin{array}{c} {}^{i}X_{0}a_{0}+v_{i}\times S_{i}\dot{q_{i}}\\ v_{i}\times^{*}I_{i}v_{i}\\ 0\\ 0 \end{array}\right],\;\mathrm{if}\;\lambda \left(i\right)\neq 0\;b_{i}=\left[\begin{array}{c} v_{i}\times S_{i}\dot{q_{i}}\\ v_{i}\times^{*}I_{i}v_{i}\\ 0\\ 0 \end{array}\right]. \end{array}$$

Remarkably, Equation (4) represents the set of linear constraints in ***d*** and, in a sense, inverse dynamic computation consists of computing ***d*** given $f_{i}^{x}$ and ${\ddot{q}}_{i}$. Our contribution is in moving away from this classical approach replacing RNEA boundary conditions with measurements coming from sensors. For this purpose, let us also define an explicit equation for measurements by indicating with ***y*** $\in R^{y}$ the values vector measured by sensors:

(5) $$Y\left(q,\dot{q}\right)d+b_{Y}\left(q,\dot{q}\right)=y$$

The structure of ***Y*** matrix depends on the number of sensors $N_{S}$ used for each link *i* as follows:

$$\begin{array}{cccc} Y & ={\left[\begin{array}{c} Y_{1}\;\cdots \;Y_{N_{B}} \end{array}\right]}^{⊤}\in R^{N_{S}\times d}, & Y_{i} & ={\left[\begin{array}{c} Y_{i,1}^{⊤}\;\cdots \;Y_{i,{N_{S}}_{i}}^{⊤} \end{array}\right]}^{⊤}\in R^{{N_{S}}_{i}\times d_{i}}, \end{array}$$

being $N_{S}=\sum_{i}{N_{S}}_{i}$ the amount of sensors. With the same methodology, the structure of the bias vector $b_{Y}$ is also defined:

$$\begin{array}{cccc} b_{Y} & ={\left[\begin{array}{c} b_{Y_{1}}\;\cdots \;b_{Y_{N_{B}}} \end{array}\right]}^{⊤}\in R^{N_{S}}, & b_{Y_{i}} & ={\left[\begin{array}{c} b_{Y_{i,1}}^{⊤}\;\cdots \;b_{Y_{i,{N_{S}}_{i}}}^{⊤} \end{array}\right]}^{⊤}\in R^{{N_{S}}_{i}}. \end{array}$$

### 3.3. Considerations on the Representation

Equation (4) is one of many possible representations of the system dynamics. A common alternative description is the one obtained with the Euler–Lagrange formalisms [12]:

$$\begin{array}{c} M\left(q\right)\ddot{q}+C\left(q,\dot{q}\right)\dot{q}+g\left(q\right)=\tau +J^{⊤}\left(q\right)f^{x}. \end{array}$$

These equations can be obtained from Equation (4) as hereafter described. First, the vector ***d*** and the columns of ***D*** should be rearranged so that they respect the following order: $a_{1}..a_{N_{B}}$, $f_{1}^{B}..f_{N_{B}}^{B}$, $f_{1}..f_{N_{B}}$, ${\ddot{q}}_{1}..{\ddot{q}}_{N_{B}}$, $f_{1}^{x}..f_{N_{B}}^{x}$ and $\tau_{1}..\tau_{N_{B}}$. The resulting ***D*** and ***b*** are:

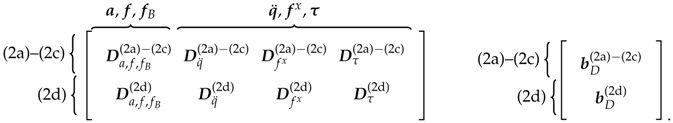


With this reorganization, the Euler–Lagrange equation can be obtained as follows:

$$\begin{array}{c} D_{a,f,f_{B}}^{\left(2d\right)}\left\{{\left[D_{a,f,f_{B}}^{\left(2a)-(2c\right)}\right]}^{-1}\left[-b_{D}^{\left(2a)-(2c\right)}-D_{\ddot{q}}^{\left(2a)-(2c\right)}\ddot{q}-D_{f^{x}}^{\left(2a)-(2c\right)}f^{x}-D_{\tau }^{\left(2a)-(2c\right)}\tau \right]\right\}+D_{\ddot{q}}^{\left(2d\right)}\ddot{q}+D_{f^{x}}^{\left(2d\right)}f^{x}+D_{\tau }^{\left(2d\right)}\tau +b_{D}^{\left(2d\right)}=0. \end{array}$$

There are two reasons for preferring Equation (4) to alternative formulations such as the Euler–Lagrange equation. On the one hand, Equation (4) can be used to represent uncertainties that capture relevant modeling approximations (see also Section 3.4). In particular, approximations result from the fact that human bones coupling is neither rigid nor purely rotational and these can be captured with additive noise Equation (9) on Equation (2). More accurate models would be possible [14], but Equation (9) would still capture approximations on bone couplings, which are often relevant and meaningful. On the other hand, there are numerical advantages associated to Equation (4). In the case of inverse and forward dynamics, the numerical advantages are exactly those obtained by algorithms like the RNEA and the ABA (Articulated-Body Algorithm) presented in [11] and whose relations to Equation (4) is discussed in [10].

### 3.4. Over Constrained RNEA

Combining properly Equations (4) and (5), we obtain:

(6) $$\begin{array}{c} \left[\begin{array}{c} Y(q,\dot{q})\\ D(q,\dot{q}) \end{array}\right]d+\left[\begin{array}{c} b_{Y}\left(q,\dot{q}\right)\\ b_{D}\left(q,\dot{q}\right) \end{array}\right]=\left[\begin{array}{c} y\\ 0 \end{array}\right] \end{array}$$

Since our main purpose is to make *WearDY* a versatile and flexible tool, we consider the incorporation of redundant (and noisy) measurements involved in the analysis. Within this new framework, there might be conditions in which Equation (6) becomes overdetermined and an exact solution does not exist. If there is a valid reason to assume that all the constraints have equal relevance, we can use the Moore–Penrose pseudo-inverse to obtain a least square solution. Otherwise, if we have good reason for weighting differently the constraints, we can use the weighted pseudo-inverse to obtain a weighted square solution. However, finding proper weights might be not an easy task. Our solution, proposed in the next section, is framing the estimation of ***d*** given ***y*** in a Gaussian framework by means of a minimum-variance estimator.

## 4. Maximum *a Posteriori* (MAP) Estimator

The first assumption for adopting the *Maximum a Posteriori* (MAP) estimation approach is to consider ***d*** and ***y*** as stochastic variables with Gaussian distributions. Let us first define their suitable joint probability density using the factorization p(d,y)=p(d)p(y|d) being p(·) the probability density and p(·|·) its conditioned version. Given p(d,y), we can compute an estimation of ***d*** using a MAP estimator (which, in Gaussian distributions, coincides with the mean of the distribution) as follows:

$$d_{MAP}=\arg\max_{d}p\left(d|y\right)\propto \arg\max_{d}p\left(d,y\right),$$

where we applied Bayes’ rule, *i.e.*, p(d|y)=p(d,y)/p(y), and where we omitted the term p(y) since it does not depend on ***d*** and does not contribute to the optimization.

Let us first give an expression for p(y|d):

$$p\left(y|d\right)∼N\left(\mu_{y},\Sigma_{y}\right),\;\mu_{y}=Y\left(q,\dot{q}\right)d+b_{Y},$$

which implicitly makes the assumption that the measurements from Equation (5) are affected by a Gaussian noise with zero mean and covariance $\Sigma_{y}$. Its probability distribution is:

(7) $$p\left(y|d\right)\propto \exp\left\{-\frac{1}{2}{\left(y-\mu_{y}\right)}^{⊤}\Sigma_{y}^{-1}\left(y-\mu_{y}\right)\right\}$$

The second assumption is to define a probability density for ***d***. Pursuing the same methodology, we would like to have the following distribution $p\left(d\right)∼N\left(\mu_{D},\Sigma_{D}\right)$ :

(8) $$p\left(d\right)\propto \exp\left\{-\frac{1}{2}e{\left(d\right)}^{⊤}\Sigma_{D}^{-1}e\left(d\right)\right\}$$

taking into account constraints in Equation (4) with $e\left(d\right)=D\left(q,\dot{q}\right)d+b_{D}$.

However, this intuitive choice leads to a degenerate normal distribution and a term of regularization has to be adopted. For example, if we have a Gaussian prior knowledge on ***d*** in the form of $p\left(d\right)∼N\left(\mu_{d},\Sigma_{d}\right)$ distribution, we can reformulate Equation (8) as $p\left(d\right)∼N\left({\bar{\mu }}_{D},{\bar{\Sigma }}_{D}\right)$:

(9) $$p\left(d\right)\propto \exp\left\{-\frac{1}{2}\left[e{\left(d\right)}^{⊤}\Sigma_{D}^{-1}e\left(d\right)+{\left(d-\mu_{d}\right)}^{⊤}\Sigma_{d}^{-1}\left(d-\mu_{d}\right)\right]\right\}$$

with

(10a) $$\begin{array}{ccc} {\bar{\Sigma }}_{D} & = & {\left(D^{⊤}\Sigma_{D}^{-1}D+\Sigma_{d}^{-1}\right)}^{-1} \end{array}$$

(10b) $$\begin{array}{ccc} {\bar{\mu }}_{D} & = & {\bar{\Sigma }}_{D}\left(\Sigma_{d}^{-1}\mu_{d}-D^{⊤}\Sigma_{D}^{-1}b_{D}\right) \end{array}$$

Given Equations (7) and (9), we can build the joint probability as $p\left(d|y\right)∼N\left(\mu_{d|y},\Sigma_{d|y}\right)$ being

(11a) $$\begin{array}{ccc} \Sigma_{d|y} & = & {\left({\bar{\Sigma }}_{D}^{-1}+Y^{⊤}\Sigma_{y}^{-1}Y\right)}^{-1} \end{array}$$

(11b) $$\begin{array}{ccc} \mu_{d|y} & = & \Sigma_{d|y}\left[Y^{⊤}\Sigma_{y}^{-1}\left(y-b_{Y}\right)+{\bar{\Sigma }}_{D}^{-1}{\bar{\mu }}_{D}\right] \end{array}$$

where Equation (11b) is exactly the estimation $d_{MAP}$.

### On the Benefits of MAP Dynamics

In this section, we discuss the benefits of multi-sensor data fusion for solving the dynamic estimation problem by characterizing the effects of data fusion on the covariance of associated estimator. The general idea we would like to pursue is that the more sensors we use in the estimation, the better the estimation itself will be (see Appendix A for the metric used for the estimation quality). Since we are interested in the analytic solution of MAP, the estimator must have the following covariance (combining Equations (11a) and (10a)):

(12) $$\begin{array}{c} \Sigma_{d|y}={\left(D^{⊤}\Sigma_{D}^{-1}D+\Sigma_{d}^{-1}+Y^{⊤}\Sigma_{y}^{-1}Y\right)}^{-1} \end{array}$$

Assuming multiple measurements $y_{1}=Y_{1}d+b_{Y_{1}}$, …, $y_{m}=Y_{m}d+b_{Y_{m}}$ statistically independent, this implies a diagonal structure to the matrix $\Sigma_{y}^{-1}$. Thus, we have:

$$\begin{array}{c} Y^{⊤}\Sigma_{y}^{-1}Y=\left[\begin{array}{ccc} Y_{1}^{⊤} & \cdots & Y_{m}^{⊤} \end{array}\right]\left[\begin{array}{ccc} \Sigma_{y_{1}}^{-1} & \cdots & 0\\ \vdots & \ddots & \vdots \\ 0 & \cdots & \Sigma_{y_{m}}^{-1} \end{array}\right]\left[\begin{array}{c} Y_{1}\\ \vdots \\ Y_{m} \end{array}\right]\;\Rightarrow \;Y^{⊤}\Sigma_{y}^{-1}Y=Y_{1}^{⊤}\Sigma_{y_{1}}^{-1}Y_{1}+\cdots +Y_{m}^{⊤}\Sigma_{y_{m}}^{-1}Y_{m}. \end{array}$$

With an abuse of notation, let us denote with $d|y_{i}$ the estimator which exploits all measurements up to *i*-th, *i.e.*, $y_{1},\cdots ,y_{i}$. The addition of one measurement induces changes in the associated covariance matrix according to the following recursive equation:

(13) $$\begin{array}{c} \Sigma_{d|y_{i}}^{-1}=\Sigma_{d|y_{i-1}}^{-1}+Y_{i}^{⊤}\Sigma_{y_{i}}^{-1}Y_{i} \end{array}$$

where, for i=1 , the initial condition is $\Sigma_{d|y_{0}}^{-1}=D^{⊤}\Sigma_{D}^{-1}D+\Sigma_{d}^{-1}$.

Considering the Weyl inequality for the largest eigenvalue $\lambda_{1}$ of Equation (13) (see Appendix B):

$$\begin{array}{c} \frac{\lambda_{N}\left(\Sigma_{d|y_{i-1}}\right)}{1+\lambda_{N}\left(\Sigma_{d|y_{i-1}}\right)\lambda_{1}\left(Y_{i}^{⊤}\Sigma_{y_{i}}^{-1}Y_{i}\right)}\leq \lambda_{1}\left(\Sigma_{d|y_{i}}\right)\leq \frac{1}{L_{1}},\;L_{1}=\max_{i+j=N+1}\left[\frac{1}{\lambda_{N-i+1}\left(\Sigma_{d|y_{i-1}}\right)}+\lambda_{j}\left(Y_{i}^{⊤}\Sigma_{y_{i}}^{-1}Y_{i}\right)\right]. \end{array}$$

The maximum benefit is obtained by lowering the upper bound on $\lambda_{1}\left(\Sigma_{d|y_{i}}\right)$. Trivially, this can be obtained by choosing high values for all the eigenvalues of $Y_{i}^{⊤}\Sigma_{y_{i}}^{-1}Y_{i}$. Obviously, this is not always possible and benefits can be obtained by maximizing $L_{1}$.

## 5. Experimental Results

### 5.1. Experimental Set-up

**Figure 2:**
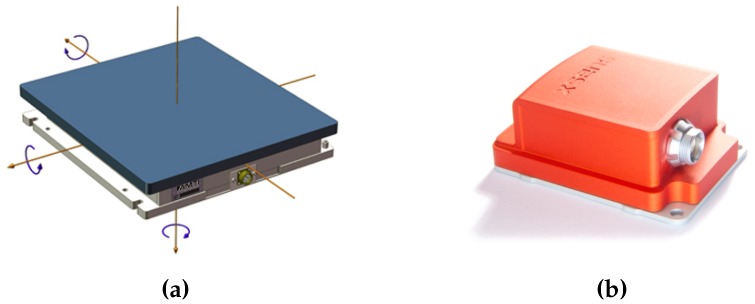
Sensors used in the experiment: a force platform AMTI OR6 (**a**) and an inertial sensor Xsens MTx comprising both accelerometer and gyroscope (**b**).

**Figure 3:**
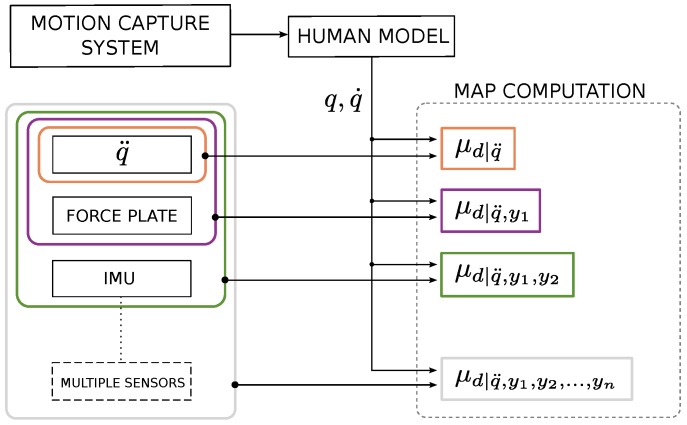
General scheme of the experimental set-up including a motion capture system and a block of sensors. Data coming from the motion system were used as inputs for each Maximum-a-Posteriori (MAP) computation and, progressively, sensor measurements ($\ddot{q}$, force plate sensing and inertial sensor IMU) were added to compute a more complete version of MAP. Although only three sensors are used (the ‘multiple sensors’ block is not present in this version of experiment), in general, the set-up can embed *n* sensors and exploit their measurements to enhance the dynamic estimation of ***d***.

Experiments have been conducted on 12 adult healthy subjects (nine males and three females). Subjects provided their written informative consent before becoming involved in the research. Motion data were collected at Istituto Italiano di Tecnologia (IIT), Genova, Italy, using a motion capture system (Vicon Motion Systems Ltd, Oxford, UK) with eight infrared cameras, at a sampling rate of 100 Hz. A total of 14 passive retro-reflective markers were attached to each participant at key anatomical landmarks. Markers are properly positioned in order to capture the pitch motion on the sagittal plane. Lower body markers are placed in the following positions: left and right back of the foot (heel), left and right outside of the ankle, left and right 2nd toe of the foot, left and right hip joint. For the upper body, they are placed on the bony prominence on top of both shoulders and one in the center of the upper torso. No arm and head motions are evaluated in this experiment. Kinematics information are recorded at a sampling rate of 100 Hz using an inertial sensor unit (Xsens Technologies B.V., Enschede, Netherlands) attached with an elastic strip on the trunk, including an accelerometer and a gyroscope (Figure 2b). Three markers are placed on the IMU external surface in order to compare data from the Vicon system and the inertial sensor. The task is performed on a standard force platform AMTI OR6 (Advanced Mechanical Technology Inc., Watertown, USA) synchronized with the Vicon system and data are recorded at a sampling rate of 1 kHz (Figure 2a). Each subject is asked to perform a bowing task without bending the knee in order to assume legs as a rigid link. Each participant experiment session consisted of four trials each composed of three bows. A general scheme of the experimental set-up is summarized in Figure 3.

### 5.2. Human Body Modeling

**Figure 4:**
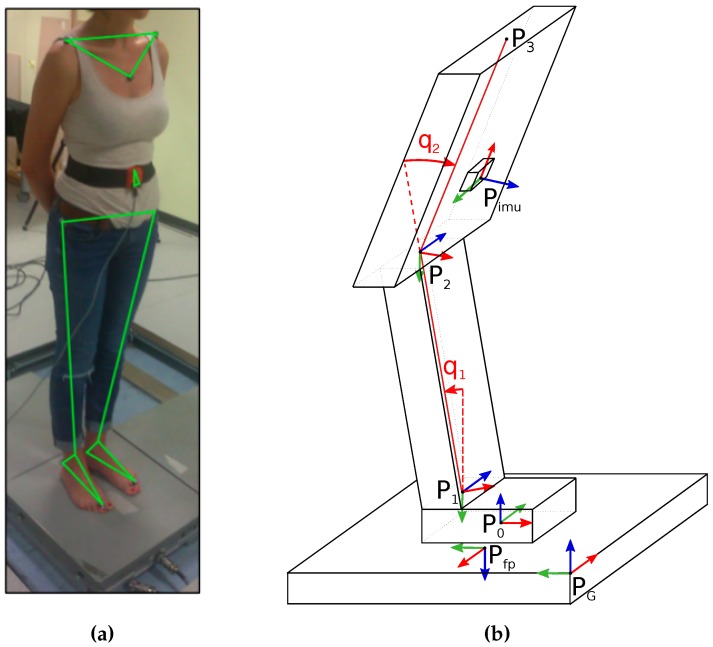
Human body modeling: generic representation of a marker-like model for a subject (**a**); its representation with reference frames associated to points (*P*_0_, *P*_1_, *P*_2_, *P*_3_) and to sensors (**b**). Reference frames are denoted by using the RGB (Red-Green-Blue) convention for *x*-*y*-*z* axis.

Inspired by state-of-the-art human stance modeling [15], the human body was modeled as a double-inverted-pendulum (DIP). A two DOF ($N_{B}=2$, $d\in R^{52}$) model of the human body for each subject was developed by using the Universal Robot Description Format (URDF) which is an XML-based file format for representing kinematics and dynamics of multibody systems. The location of four imaginary points in the body ($P_{0},P_{1},P_{2},P_{3}$) are computed by averaging the location of Vicon markers. The final model consists of three rigid links representing the feet (link 0 or fixed base), legs (link 1) and torso (link 2) and two revolute joints positioned at ankle (joint 1) and hip (joint 2) and subjected to gravity. Points $P_{0},P_{1},P_{2},P_{3}$ represent the origin of the reference frames associated to each link (with the exception for $P_{3}$ as link 3 does not exist). In particular, reference frames have the same orientation of the body to which they are associated. No rigid links for head or arms were considered. Figure 4 shows human modeling passing from a marker-like Figure 4a to a URDF-like model Figure 4b.

### 5.3. Proof of Concept

**Figure 5:**
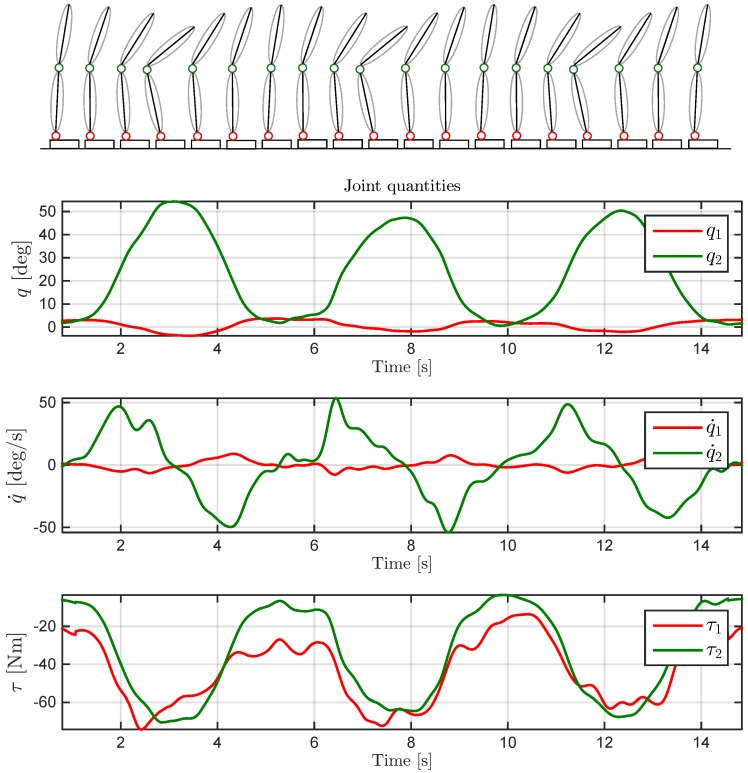
Representation of a trial composed of three repetitive bows (**on top**). Joint quantities: angles $q_{1}$ and $q_{2}$ have been obtained from motion capture acquisition with a frequency filtering; velocities ${\dot{q}}_{1}$ and ${\dot{q}}_{2}$ have been computed using a third-order polynomial Savitzky–Golay filtering; torques have been computed using MAP estimation exploiting all sensors involved in the analysis (**on bottom**).

Analysis is performed on MATLAB using a toolbox released by Roy Featherstone [16] and freely available under an open source licence. The core of the experiment consists in using the MAP algorithm to estimate for both links in the model the dynamic vectors $d_{1}\left(a_{1},f_{1}^{B},f_{1},\tau_{1},f_{1}^{x},{\ddot{q}}_{1}\right)$ and $d_{2}\left(a_{2},f_{2}^{B},f_{2},\tau_{2},f_{2}^{x},{\ddot{q}}_{2}\right)$. In the proposed experiment, $q={\left[q_{1}\;q_{2}\right]}^{⊤}$ was obtained from Vicon marker analysis with a frequency filtering in order to smooth acquisition noise. Joint velocities $\dot{q}={\left[{\dot{q}}_{1}\;{\dot{q}}_{2}\right]}^{⊤}$ and accelerations $\ddot{q}={\left[{\ddot{q}}_{1}\;{\ddot{q}}_{2}\right]}^{⊤}$ have been computed using a weighted sum of windows of elements, with a third-order polynomial Savitzky–Golay filtering (as shown in Figure 5). Considering that during the experiment no external force was applied, $f^{x}={\left[f_{1}^{x}\;f_{2}^{x}\right]}^{⊤}$ was null and was simulated by associating a null measurement. Above-obtained variables and the dynamic model were used in building the constraint Equation (4).

Similarly, from the sensor equations :

$$\begin{array}{ccc} y_{1,fp} & = & {}^{fp}X_{0}^{*}\left({}^{0}X_{1}^{*}f_{1}-I_{0}{}^{0}a_{g}\right),\\ y_{2,acc} & = & {\left({}^{imu}X_{2}a_{2}\right)}_{lin}+{\left({}^{imu}X_{2}v_{2}\right)}_{ang}\times {\left({}^{imu}X_{2}v_{2}\right)}_{lin},\\ y_{2,gyro} & = & {\left({}^{imu}X_{2}v_{2}\right)}_{ang}, \end{array}$$

we built the measurements Equation (5) for three different cases: (1) $y=[\ddot{q}]$; (2) $y={\left[\ddot{q}\;y_{1}\right]}^{⊤}$; (3) $y={\left[\ddot{q}\;y_{1}\;y_{2}\right]}^{⊤}$. It is worth noticing that the gyroscope measurement has been numerically differentiated to obtain a measurement for the angular part of $a_{i}$ (*i.e.*, ${\dot{\omega }}_{i}$). Since the final MAP estimation $\mu_{d|y}$ is built as a weighted sum of *a priori* distribution and sensor measurements, it has required defining $\Sigma_{D}$ and $\Sigma_{d}$ (in order to establish how much the dynamic model was reliable) and $\Sigma_{y}$ to properly weight the contribution of each sensor. In the present analysis, the standard deviations *σ* have been chosen from sensors’ datasheets, and their values are reported in Table 1.

**Figure 6:**
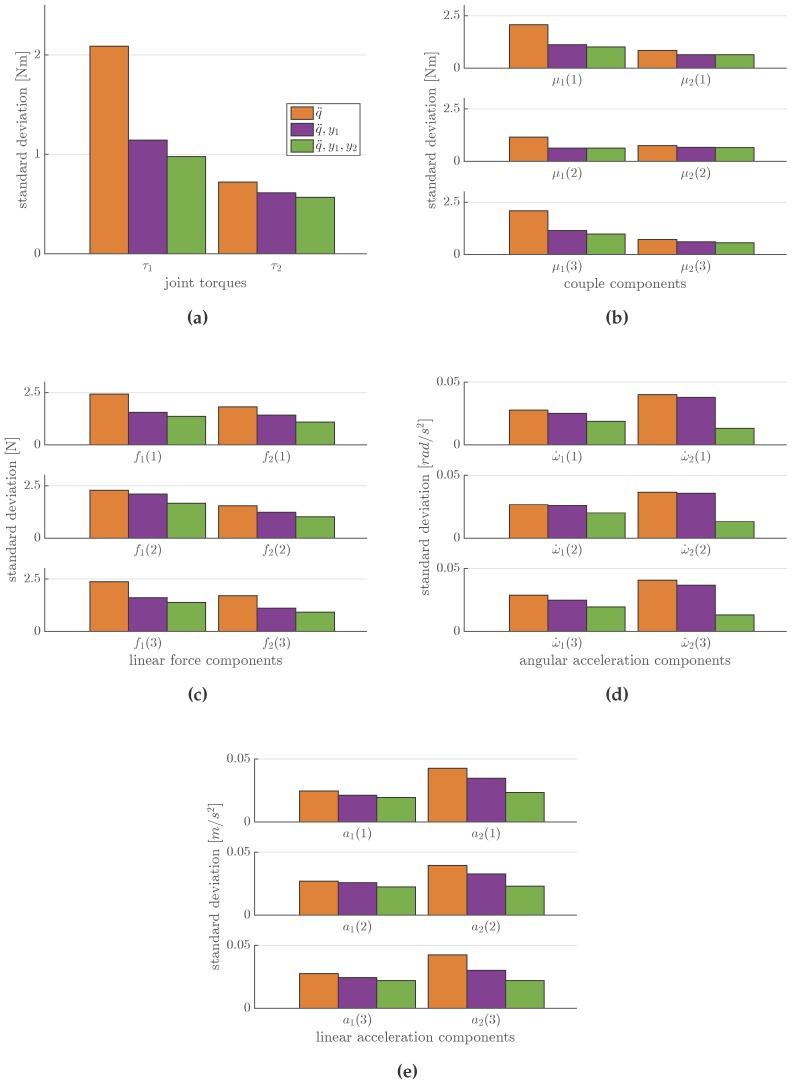
Considering three MAP computation cases with one sensor ($y=\ddot{q}$), two sensors ($y=\ddot{q},y_{1}$), and all sensors ($y=\ddot{q},y_{1},y_{2}$), we computed standard deviation of (**a**) $\Sigma_{\tau_{1}|y}$ and $\Sigma_{\tau_{2}|y}$ ; (**b**,**c**) $\Sigma_{f_{1}|y}$ and $\Sigma_{f_{2}|y}$ for both angular (μ) and linear (***f***) components; (**d**,**e**) $\Sigma_{a_{1}|y}$ and $\Sigma_{a_{2}|y}$ for both angular ($\dot{\omega }$) and linear (***a***) components. These plots show that when increasing the number of sensors in the analysis, the standard deviation of each variable estimated in the vector $\mu_{d|y}$ progressively decrease.

**Figure 7:**
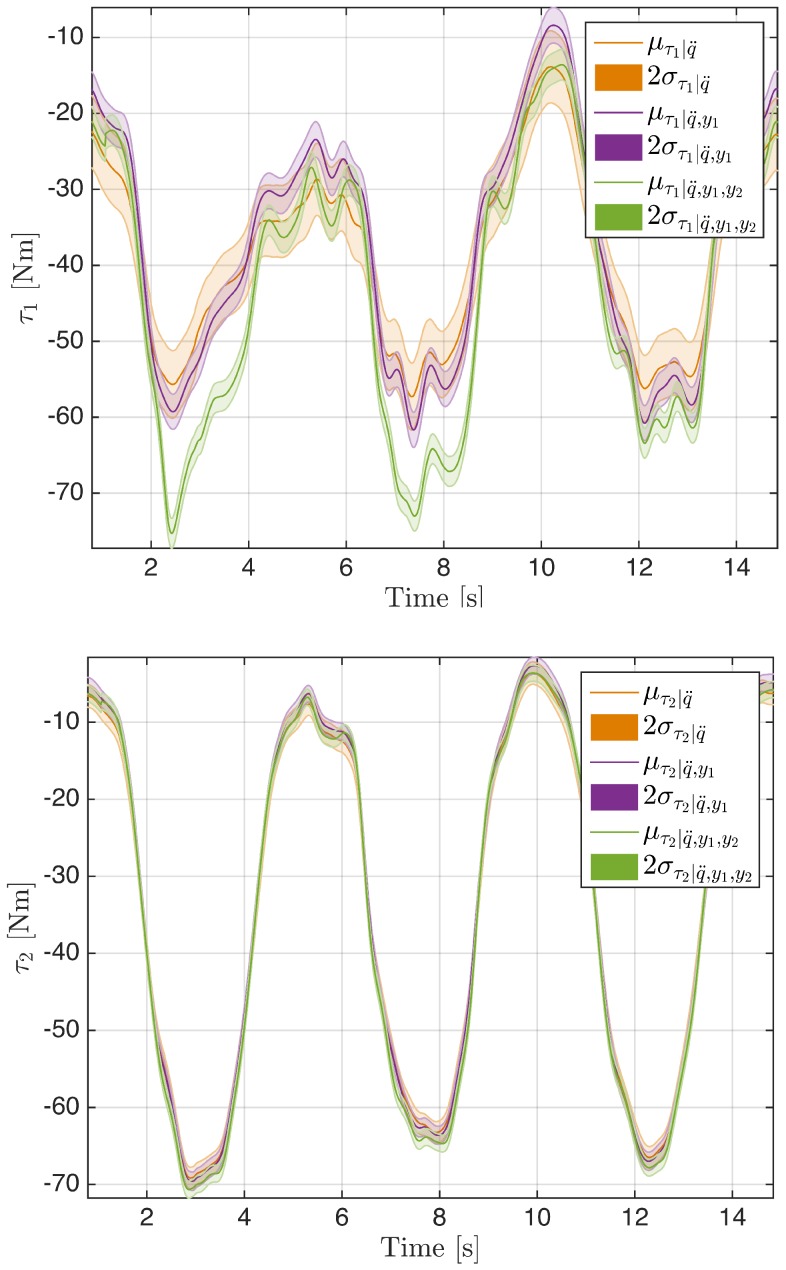
Mean (*μ*) and standard deviation (2*σ*) for the estimation of τ increasing the number of sensors.

The experiment was conducted to prove exactly what was described in Section 4.1: passing progressively from sensor *case 1* to *case 3*, the variance associated to each variable in the estimated vector $\mu_{d|y}$ decrease. Figure 6 shows the *σ* decreasing behavior for three variables ($\sigma_{\tau |y}$, $\sigma_{f|y}$ and $\sigma_{a|y}$) using MAP computation as sensors have risen. Variance data are reported in Table 2 for link 1 and Table 3 for link 2. In Figure 7, we focused on the estimated $\mu_{\tau |y}$ for both links in the model. Estimations are represented with their standard deviations (2*σ*). It is worth noticing that, while the estimations for $\tau_{2}$ are very comparable, estimations for $\tau_{1}$ are less similar due to the fact that, in a sense, $\tau_{1}$ is almost a direct measurement to compensate for the model error in the computation.

### 5.4. Method Robustness Test

This section describes the analysis we performed to test the robustness w.r.t. modeling errors of the MAP and RNEA. As in the previous experiments, we performed a bowing task. This time, the subject repeated the experiment in two different configurations, that is *with* and *without* an additional weight of 5 kg roughly positioned in correspondence of the center of mass (COM) of the torso. By exploiting the linearity property of the system, we started by considering the expression for the torques:

(15) $$\begin{array}{c} \tau_{\left(model+5kg\right)}-\tau_{\left(model\right)}=\tau_{\left(5kg\right)} \end{array}$$

where we denoted with *model+5kg* the kinematic and dynamic model of the subject with the additional 5 kg mass on the torso, and with *model* the model of the subject without the weight. The above expression exploits the property of linearity of the model w.r.t. a set of dynamic parameters [12], and it is in general described by the following equation:

$$\begin{array}{c} \tau =Y(q,\dot{q},\ddot{q})\pi , \end{array}$$

where π is a vector of constant parameters and ***Y***, usually known as *regressor*, is the matrix function of joint positions, velocities and accelerations.

To assess the robustness property of the two algorithms w.r.t. modeling errors, we then computed the inverse dynamics by using, respectively, RNEA and MAP, considering both the measurements collected while the subject was performing the task with the 5 kg weight ($y_{\text{subject}+5\mathrm{kg}}$) and the measurements during the task without the weight ($y_{\text{subject}}$), as follows:

(16) $$\begin{array}{cc} & \tau_{RNEA(model,y_{subject+5kg})}-\tau_{RNEA(model,y_{subject})}={\hat{\tau }}_{RNEA(5kg)} \end{array}$$

(17) $$\begin{array}{cc} & \tau_{MAP(model,y_{subject+5kg})}-\tau_{MAP(model,y_{subject})}={\hat{\tau }}_{MAP(5kg)} \end{array}$$

**Figure 8:**
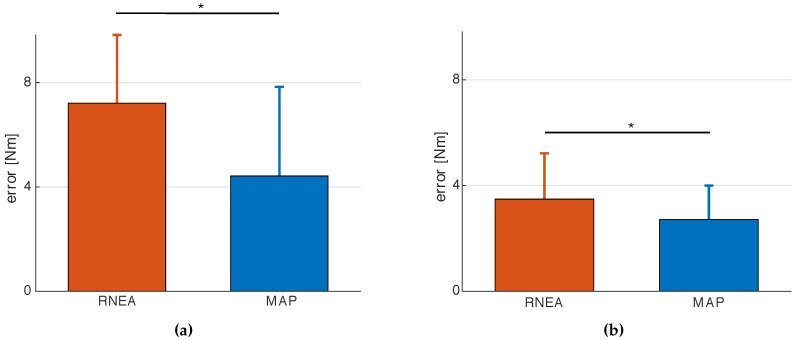
Error on torque estimation comparing RNEA and MAP methods on $\tau_{1}$ (**a**) and $\tau_{2}$ (**b**), (* *p*-value < 0.05).

Then, we compare the right-hand side of Equations (16) and (17) with the right-hand one of Equation (15). Figure 8 show mean and standard deviation for the error, respectively, on $\tau_{1}$ and $\tau_{2}$ comparing $\left|\tau_{\left(5kg\right)}-{\hat{\tau }}_{RNEA(5kg)}\right|$ (on the left part of each figure) and $\left|\tau_{\left(5kg\right)}-{\hat{\tau }}_{MAP(5kg)}\right|$ (on the right part). It is worth noticing that the error on the estimation of $\tau_{2}$ is statistically significant different (*p*-value < 0.05) because $\tau_{2}$ is more influenced by the additional weight on the torso. It is also worth remarking that, in order to compare the obtained torques in the different trials, we expressed them w.r.t. a linear combination of configuration variables instead of expressing them w.r.t. the time. Indeed, it was not possible to evaluate, in time *t*, the comparison between torques of different trials because the same subject performed the trials in different times; thus, the estimation of $\tau_{2}$ has been parameterized with a linear combination of the joint positions, *i.e.*, $\left(q_{1}+q_{2}\right)$, with the primary assumption that $\dot{q}$ and $\ddot{q}$ could be neglected as they induced a small change on the τ estimation. We can observe that the error on the τ estimation is lower in MAP than in RNEA since our procedure is able to represent the model uncertainties. From this point of view, RNEA is, in a sense, more sensitive to the modeling errors. Conversely, MAP takes into account these errors because a variance is also associated to the model itself.

## 6. Conclusions

In this paper, we presented a novel methodology to estimate dynamics quantities along with human motion in order to exploit fusion of sensor information on a probabilistic framework. The framework is based on the idea of building a joint probability for all dynamic variables ***d*** and measurements ***y*** coming from multiple sensors. The probability density p(d,y) incorporates the dynamic constraints among variables. Preliminary results show that adding the *i*-th measurement induces changes in its covariance matrix according to Equation (13) for all variables estimated. We demonstrate that the variance of each estimated variable in $\mu_{d|y}$ decreases as the number of considered measurements (sensors) increase. Moreover, it should be necessary to implement a procedure (Expectation-Maximization (EM) algorithm) to estimate sensor variance from data; in a sense, the goal is to build a *data-driven* variance estimation overcoming datasheet variance. While this paper presents a proof of concept of the theory behind *WearDY*, future works will be aimed at real-time implementation, at the design of the wearable garment and at the improvement of the sensor architecture in order to make the system reliable *in situ*. Computationally, it will be fundamental to integrate MAP dynamics with a state estimator such as an Extended Kalman filter combining obtained *a posteriori* estimates with *a priori* estimates of the filter state. From a design perspective, the wearable system will be comprised of a soft sensing garment with embedded sensors that facilitate free movement of the subject. The suit design will ideally exploit the material compliance in order to improve sensor reliability through elimination of the error introduced by the interface between the garment and the subject’s skin. Another important future objective concerns the possibility of using electromyography (EMG) analysis to provide information on muscular activity that is related to joint torques. Possible future applications of *WearDY* include rehabilitation monitoring where a wearable garment will be used for monitoring patients or creating more ergonomic and compliant prosthesis and exoskeleton systems. The proposed suit and the forthcoming improvements would permit substantial enhancements in the analysis of human movements in motion.

## Figures and Tables

**Table 1:** Standard deviation *σ* from sensors’ datasheets.

	Angular Part			Linear Part		
$\sigma_{\ddot{q}}$				$2.57\times 10^{-3}\;\mathrm{rad}/s^{2}$		
$\sigma_{y_{1}}$	1.5 Nm	1.5 Nm	0.7483 Nm	7.68 N	7.68 N	6 N
$\sigma_{y_{2}}$	$7.1\times 10^{-3}\;\mathrm{rad}/s^{2}$ (gyro)			$3.33\times 10^{-2}\;m/s^{2}$ (acc)		

**Table 2:** Standard deviation of estimated variables for link 1.

	$\sigma_{\tau_{1}}$	$\sigma_{\mu_{1}\left(1\right)}$	$\sigma_{\mu_{1}\left(2\right)}$	$\sigma_{\mu_{1}\left(3\right)}$	$\sigma_{f_{1}\left(1\right)}$	$\sigma_{f_{1}\left(2\right)}$	$\sigma_{f_{1}\left(3\right)}$	$\sigma_{{\dot{\omega }}_{1}\left(1\right)}$	$\sigma_{{\dot{\omega }}_{1}\left(2\right)}$	$\sigma_{{\dot{\omega }}_{1}\left(3\right)}$	$\sigma_{a_{1}\left(1\right)}$	$\sigma_{a_{1}\left(2\right)}$	$\sigma_{a_{1}\left(3\right)}$
$\ddot{q}$	4.990	2.075	1.153	2.088	2.423	2.279	2.371	0.028	0.027	0.029	0.025	0.027	0.028
$\ddot{q},y_{1}$	1.340	1.118	0.636	1.145	1.558	2.099	1.600	0.025	0.026	0.025	0.021	0.026	0.024
$\ddot{q},y_{1},y_{2}$	0.970	1.020	0.633	0.977	1.364	1.659	1.382	0.019	0.020	0.019	0.019	0.022	0.022

**Table 3:** Standard deviation of estimated variables for link 2.

	$\sigma_{\tau_{2}}$	$\sigma_{\mu_{2}\left(1\right)}$	$\sigma_{\mu_{2}\left(2\right)}$	$\sigma_{\mu_{2}\left(3\right)}$	$\sigma_{f_{2}\left(1\right)}$	$\sigma_{f_{2}\left(2\right)}$	$\sigma_{f_{2}\left(3\right)}$	$\sigma_{{\dot{\omega }}_{2}\left(1\right)}$	$\sigma_{{\dot{\omega }}_{2}\left(2\right)}$	$\sigma_{{\dot{\omega }}_{2}\left(3\right)}$	$\sigma_{a_{2}\left(1\right)}$	$\sigma_{a_{2}\left(2\right)}$	$\sigma_{a_{2}\left(3\right)}$
$\ddot{q}$	0.510	0.848	0.758	0.758	1.817	1.544	1.696	0.040	0.037	0.041	0.043	0.040	0.042
$\ddot{q},y_{1}$	0.356	0.650	0.669	0.615	1.423	1.238	1.100	0.038	0.036	0.037	0.035	0.033	0.030
$\ddot{q},y_{1},y_{2}$	0.305	0.643	0.657	0.568	1.092	1.026	0.918	0.013	0.013	0.013	0.023	0.023	0.022

## Appendix A

Given two estimators ${\hat{d}}_{1}$ and ${\hat{d}}_{2}$, the first is better than the second if:

$$\begin{array}{c} \lambda_{1}\left(\Sigma_{{\hat{d}}_{1}}\right)<\lambda_{1}\left(\Sigma_{{\hat{d}}_{2}}\right), \end{array}$$

$\Sigma_{x}$ being the covariance matrix of the stochastic variable ***x*** and $\lambda_{1}\left(A\right)$ the maximum eigenvalue of the matrix ***A***. The motivation behind this choice lies in the fact that the maximum eigenvalue bounds the variance of any other linear combination of ***d***. Let us assume that we are interested in estimating $x=v^{⊤}d$ by choosing $\hat{x}=v^{⊤}\hat{d}$. The estimator variance is given by:

$$\begin{array}{c} \sigma \left(\hat{x}\right)=v^{⊤}\Sigma_{\hat{d}}\;v\leq {∥v∥}^{2}\cdot \lambda_{1}\left(\Sigma_{\hat{d}}\right), \end{array}$$

with the inequality being an identity when ***v*** equals the eigenvector associated to the maximum eigenvalue.

## Appendix B

Let A,B be Hermitian $\in R^{N\times N}$ matrices, $A=A^{⊤}>0$ and $B=B^{⊤}\geq 0$; thus, we have A+B>0. Let λ(A),λ(B) be eigenvalues of ***A*** and ***B***, respectively. Consider also an eigenvalue decreasing numbering for each matrix, from the largest to the smallest one. We can apply the Weyl inequality being *k* the *k*-th largest eigenvalue as follows:

(B1) $$\begin{array}{c} L_{k}\leq \lambda_{k}\left(A+B\right)\leq U_{k} \end{array}$$

$$\begin{array}{c} L_{k}=\max_{i+j=1+N}\left[\lambda_{i}\left(A\right)+\lambda_{j}\left(B\right)\right],\;U_{k}=\min_{i+j=1+k}\left[\lambda_{i}\left(A\right)+\lambda_{j}\left(B\right)\right], \end{array}$$

where $L_{k}$ and $U_{k}$ are the lower and the upper bounds, respectively. Without loss of consistency, let us assume k=1; thus, Equation (B1) becomes:

(B2) $$\begin{array}{c} L_{1}\leq \lambda_{1}\left(A+B\right)\leq U_{1} \end{array}$$

$$\begin{array}{c} L_{1}=\max_{i+j=1+N}\left[\lambda_{i}\left(A\right)+\lambda_{j}\left(B\right)\right],\;U_{1}=\min_{i+j=1+1}\left[\lambda_{i}\left(A\right)+\lambda_{j}\left(B\right)\right]. \end{array}$$

If ***A*** and ***B*** are non-singular matrices and $\lambda_{1}\neq 0$, then $\frac{1}{\lambda_{1}}$ is an eigenvalue of $A^{-1}$. Thus, in Equation (B2):

(B3) $$\begin{array}{c} \frac{1}{\lambda_{1}\left(A\right)+\lambda_{1}\left(B\right)}\leq \lambda_{1}\left({\left(A+B\right)}^{-1}\right)\leq \frac{1}{L_{1}},\;\;L_{1}=\max_{i+j=N+1}\left[\lambda_{i}\left(A\right)+\lambda_{j}\left(B\right)\right] \end{array}$$

Substituting in Equation (B3) $A=\Sigma_{d|y_{i-1}}^{-1}$ and $B=Y_{i}^{⊤}\Sigma_{y_{i}}^{-1}Y_{i}$:

$$\begin{array}{c} \frac{1}{\lambda_{1}\left(\Sigma_{d|y_{i-1}}^{-1}\right)+\lambda_{1}\left(Y_{i}^{⊤}\Sigma_{y_{i}}^{-1}Y_{i}\right)}\leq \lambda_{1}\left(\Sigma_{d|y_{i}}\right)\leq \frac{1}{L_{1}},\;L_{1}=\max_{i+j=N+1}\left[\lambda_{i}\left(\Sigma_{d|y_{i-1}}^{-1}\right)+\lambda_{j}\left(Y_{i}^{⊤}\Sigma_{y_{i}}^{-1}Y_{i}\right)\right]. \end{array}$$

Since $\lambda_{i}\left(\Sigma_{d|y_{i-1}}^{-1}\right)=\frac{1}{\lambda_{N-i+1}\left(\Sigma_{d|y_{i-1}}\right)}$ :

$$\begin{array}{c} \frac{\lambda_{N}\left(\Sigma_{d|y_{i-1}}\right)}{1+\lambda_{N}\left(\Sigma_{d|y_{i-1}}\right)\lambda_{1}\left(Y_{i}^{⊤}\Sigma_{y_{i}}^{-1}Y_{i}\right)}\leq \lambda_{1}\left(\Sigma_{d|y_{i}}\right)\leq \frac{1}{L_{1}},\;L_{1}=\max_{i+j=N+1}\left[\frac{1}{\lambda_{N-i+1}\left(\Sigma_{d|y_{i-1}}\right)}+\lambda_{j}\left(Y_{i}^{⊤}\Sigma_{y_{i}}^{-1}Y_{i}\right)\right]. \end{array}$$
